# Precision medicine based on the phenotypic differences in peripheral T helper cells in patients with psoriatic arthritis: One year follow-up outcomes

**DOI:** 10.3389/fmed.2022.934937

**Published:** 2022-07-27

**Authors:** Ippei Miyagawa, Shingo Nakayamada, Masanobu Ueno, Yusuke Miyazaki, Naoaki Ohkubo, Yoshino Inoue, Satoshi Kubo, Yoshiya Tanaka

**Affiliations:** The First Department of Internal Medicine, School of Medicine, University of Occupational and Environmental Health, Kitakyushu, Japan

**Keywords:** psoriatic arthritis, precision medicine, biological DMARDs, treatment, peripheral T lymphocyte phenotyping

## Abstract

**Purpose:**

We validated the one-year effectiveness of strategic treatment with biological disease-modifying anti-rheumatic drugs (bDMARDs) based on peripheral T-lymphocytic phenotyping and explored the impact of treatment on T helper lymphocytic phenotypes.

**Methods:**

Ninety-seven patients were registered in this study. One-year treatment response was compared between the two groups: the strategic bDMARDs treatment group (*n* = 41), in which bDMARDs were selected based on peripheral blood lymphocyte analysis, and the standard bDMARDs treatment group (*n* = 56), in which the patients underwent no strategic selection of bDMARDs and phenotyping. Changes in helper T lymphocytic phenotypes were evaluated after 1-year post-treatment.

**Results:**

In the standard bDMARDs treatment group, 23 patients (42.6%) achieved disease activity in psoriatic arthritis (DAPSA)-remission (REM), and 23 of 46 (50.0%) achieved PASI 90. In the strategic bDMARDs treatment group, 22 (53.7%) achieved DAPSA-REM, and 26 of 35 (74.2%) achieved PASI90. The rate of achieving minimal disease activity (MDA) and DAPSA-REM at month 6, DAPSA-low disease activity (LDA) at months 6 and 12, and PASI 90 at month 12 were significantly higher in the strategic bDMARDs treatment group. After treatment with ustekinumab, the proportion of aTh1/CD4 (%) significantly decreased. The percent reduction in activated Th17 cells was significantly higher in IL-17-i cells than in UST/TNF-i cells.

**Conclusions:**

The results of this study demonstrate the 1-year effectiveness of precision medicine based on peripheral T-lymphocytic phenotyping in terms of DAPSA and MDA. Analysis of data from real-world clinical practice showed that the impact on the immune system varied among bDMARDs. However, because psoriatic arthritis has very high heterogeneity, it may be necessary to conduct studies with a larger sample size, perhaps drawing samples from multiple institutions.

## Introduction

Psoriatic arthritis (PsA) is the chronic and progressive inflammatory arthritis and often results in permanent joint damage and disability. Various pro-inflammatory cytokines, such as tumor necrosis factor (TNF)-α, interleukin (IL)-23, IL-17A/F, interferon (IFN)-γ and granulocyte–macrophage colony-stimulating factor (GM-CSF) play an important role in the pathogenesis of PsA (1). European League Against Rheumatism (EULAR) recommends that use of targeted therapies, such as TNF-inhibitors (TNF-i), IL-17-inhibitors (IL-17-i), IL-12/23-inhibitors (IL-12/23-i), Janus kinase inhibitors (JAK-i), and phosphodiesterase 4 inhibitors (PDE4-i), should be considered, particularly in patients with an inadequate response to at least one conventional synthetic DMARDs (csDMARD-IR) (2). Although various drugs with distinct targets are available, the strategy for selecting the optimal biological DMARDs (bDMARDs) or JAK-i in individual cases have not been established.

Precision medicine has been recently emerging to improve treatment strategy and its outcomes for not only patients with malignancy but also systemic autoimmune diseases or rheumatic diseases including PsA which are molecularly and clinically heterogeneous (3). We previously reported the possibility of precision medicine in PsA, based on the stratification of patients with PsA, using peripheral helper T cells phenotyping (4). When bDMARDs were strategically selected based on peripheral helper T cells phenotyping in patients with PsA, the ratio of achieving SDAI (simplified disease activity index)-low disease activity after 6 months treatment was significantly higher than those who were treated with bDMARDs without flow cytometry and just following the EULAR recommendations 2015 (5). However, the observation period in that study was relatively short (6 months). Moreover, we developed treatment strategies based on the treatment target of SDAI instead of disease activity in psoriatic arthritis (DAPSA) or MDA. Therefore, in the present retrospective study, the effectiveness of our treatment strategies was validated over a 12-month observation period, based on DAPSA and MDA. In addition, the impact of bDMARDs treatment on T helper lymphocytic phenotypes was analyzed.

## Methods

### Patients and clinical measurements

**Figure 1:**
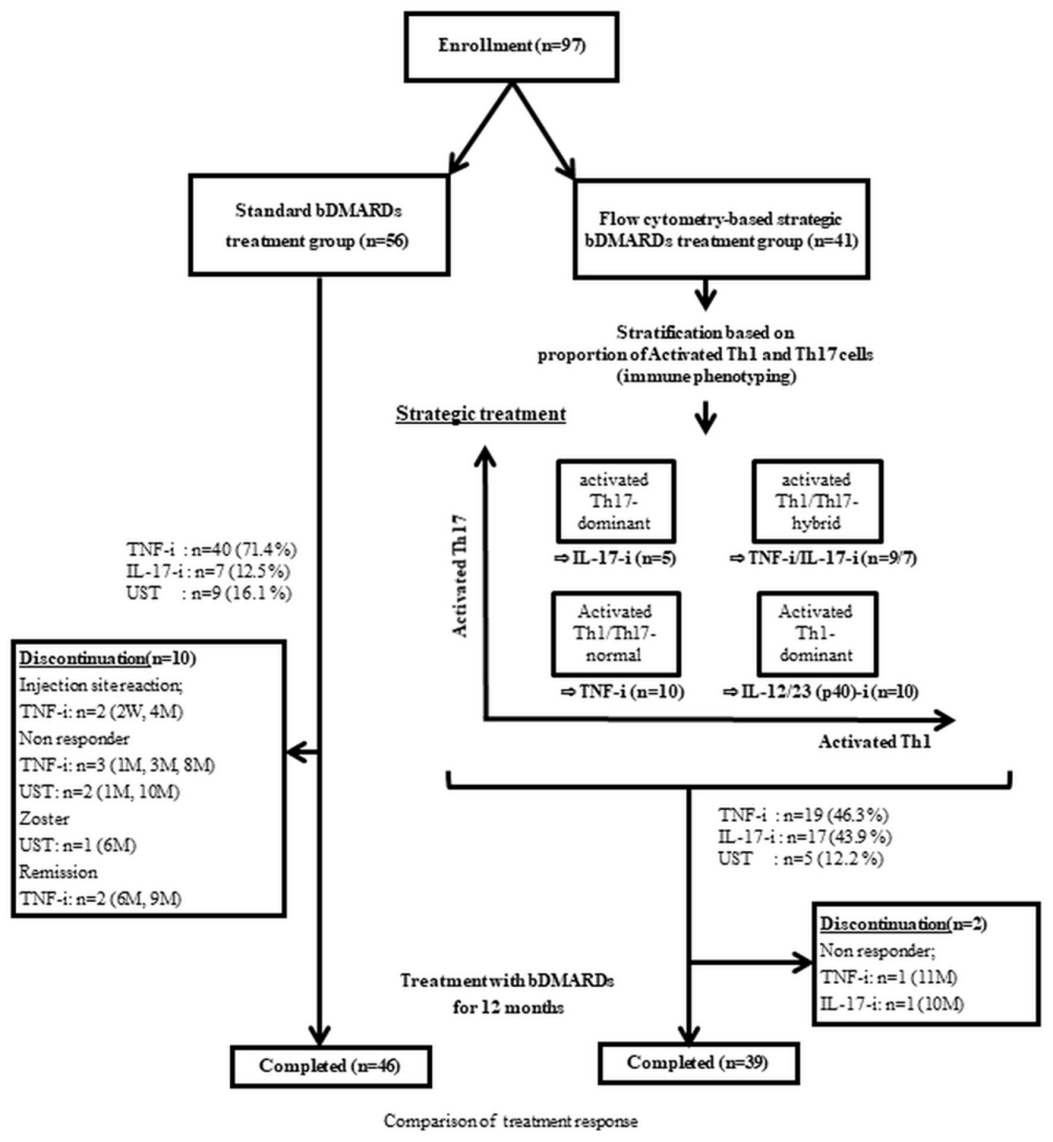
Study design. UST, ustekinumab; IL-17-I, IL-17 inhibitors; TNF-I, TNF inhibitors-index; aTh1, activated Th1 cells; aTh17, activated Th17 cells.

Ninety-seven patients with PsA diagnosed according to the CASPAR criteria and who received bDMARDs (UST [ustekinumab], TNF-i [TNF-inhibitors], and IL-17-I [IL-17-inhibitors]) for 1 year or more, at any time between 2012 and 2021, were registered in this study. All 97 patients had peripheral arthritis. One-year treatment response was compared between the two groups: the strategic bDMARDs treatment group (*n* = 41), in which bDMARDs were selected based on peripheral helper T cells phenotyping, and the standard bDMARDs treatment group (*n* = 56), in which the patients underwent no strategic selection of bDMARDs and phenotyping. Changes in helper T lymphocytic phenotypes were evaluated after 1-year post-treatment (Figure 1). Among patients who were treated at our institution in or after August 2014, when we started lymphocytic phenotyping (peripheral helper T cells phenotyping) by flow cytometry for PsA, those who consented to the initiation of strategic treatment were included in the strategic bDMARDs treatment group. The standard bDMARDs treatment group consisted of patients who were treated before August 2014, including both patients who did not consent to lymphocytic phenotyping and patients who consented to lymphocytic phenotyping but did not consent to the initiation of strategic treatment.

The treatment effects were assessed based on DAPSA (disease activity in psoriatic arthritis), (DAPSA), PASI (psoriasis area, and severity index). In addition, rate of achieving MDA (Minimal Disease Activity) were evaluated at month 6 and 12. Six patients in the strategic treatment group and ten in the standard treatment group without skin manifestations (PASI 0 at baseline) were excluded from the analysis of PASI response rates (PASI 90, % improvement of PASI). Adverse events were defined as new events or unexpected worsening of a medical condition, irrespective of cause, during the observation period. Severity of adverse events was classified according to the National Cancer Institute Common Terminology Criteria for Adverse Events (CTCAE), version 5.0.

### Treatment strategy based on phenotypic differences in peripheral helper T cells

The details of the treatment strategy are described in a previous study (4). As previously reported, UST was used for patients with the activated Th1-dominant type (activated Th1 >1.5% and activated Th17 <1.2%). IL-17-i was used in patients with activated Th17-dominant type (activated Th1 <1.5% and activated Th17 >1.2%) and for patients with activated Th1/Th17-hybrid type (activated Th1/Th1-both high type) (activated Th1 >1.5% and activated Th17 >1.2%) and major skin complaints (PASI >10). TNF-i was used for patients with activated Th1/Th17-hybrid type (activated Th1/Th1-both high type) (activated Th1 >1.5% and activated Th17 >1.2%) and major joint complaints (moderate or high disease activity) and for patients with activated Th1/Th17-comparable (to healthy control) type (activated Th1/Th1-both low type) (activated Th1 <1.5% and activated Th17 <1.2%) (4) (Figure 1). In the standard treatment group, the choice of bDMARDs was made by shared decision making between each doctor and patient, following the EULAR 2015 or the 2019 recommendation (i.e., in principle, TNF inhibitors first) (2, 5).

### Flow cytometric analysis

The details of the gating strategy are described in a previous study (2). The phenotype of helper T cells subsets was defined based on the HIP protocol of comprehensive 8-color flow cytometric analysis proposed by NIH/FOCIS (6). Immunophenotyping was conducted using multicolor flow cytometry. All collected samples were immediately analyzed using flow cytometry. Peripheral helper T cells phenotyping was performed at baseline (41 cases) and at month 6 (30 cases) and month 12 (26 cases) in the strategic bDMARDs treatment group. The cells were stained with the indicated antibodies and then analyzed through multicolor flow cytometry (FACSVerse; BD) and using the FlowJo software (Tree Star).

### Statistical analysis

Data are expressed as mean ± standard deviation (SD), median (interquartile range [IQR]), or percentage (%). Differences between groups were compared using the Mann-Whitney U test, Kruskal-Wallis test, Fisher's exact test, or chi-square test. The Wilcoxon signed-rank test was used to identify statistically significant differences between the baseline data and data at each observation point (month 6 and 12). The level of significance was set at *P* < 0.05. All analyses were performed using JMP Pro version 15 (SAS Institute Inc., Cary, NC, USA) and GraphPad Prism 9 (GraphPad Software, San Diego, CA, USA).

## Results

### Comparison of treatment response between standard and strategic BDMARDs treatment groups

In Table 1, we present the baseline characteristics. In this retrospective study, there were statistically significant differences in the smoking history, the concomitant MTX use, and initiation of bDMARDs (TNF-i and IL-17-i); however, disease activity did not differ between 2 groups. One year after bDMARD treatment, disease activity significantly improved. In the standard bDMARDs treatment group, 23 patients (42.6%) achieved DAPSA-REM (remission), and 23 of 46 patients (50.0%) achieved PASI90. In the strategic bDMARDs treatment group, 22 patients (53.7%) achieved DAPSA-REM (remission), and 26 of 35 patients (74.2%) achieved PASI90. In the comparison of treatment response between the two groups, the achieving rate of MDA and DAPSA-REM at month 6, DAPSA-LDA at months 6 and 12, and PASI 90 at month 12 were significantly higher in the strategic bDMARDs treatment group (Table 2).

**Table 1:** Differences in baseline characteristics between strategic treatment group and the standard treatment group.

**Variables**	**Standard treatment group (*n =* 56)**	**Strategic treatment group (*n =* 41)**	***p*** **-value**
Age (years)	48.3 ± 15.8	55.7 ± 15.1	*0.0076
Male, *n* (%)	26(46.4)	25(60.9)	0.6938
BMI	23.6(21.3, 27.6)	24.7(21.3, 27.6)	0.7259
Smoking history	20(35.7)	255(61.0)	0.0229
Current smoker	7(12.5)	10(24.4)	0.1770
**Disease duration (months)**			
PSO(M)	144(34.5, 201.3)	120(22, 290.5)	0.6480
PsA(M)	53(18.5, 125.8)	36(7, 123)	0.2280
Peripheral arthritis, *n* (%)	56(100)	41(100)	1.0000
Spinal involvement, *n* (%)	10(17.9)	10(24.4)	0.4565
Bio naive	31(75.6)	35(63.6)	0.2676
Concomitant MTX use	43(76.8)	22(53.7)	*0.0028
Concomitant drug use (Other than MTX)	5(8.93) PSL:*n =* 1, CsA:*n =* 2, SASP:*n =* 2	5(12.2) PSL:*n =* 1, Apremilast:*n =* 1, CsA:*n =* 4	0.7386
**bDMARDS initiated**			
IL-17-i	7(12.5)	17(43.9)	* <0.0001
TNF-i	40(71.4)	19(46.3)	*0.0200
UST	9(16.1)	5(12.2)	0.7716
**Disease activity**			
TJC (68)	6(3, 10.75)	6(2, 9.5)	0.4596
SJC (66)	4(1, 8.75)	3(1, 6)	0.5640
Pain VAS (cm)	5.25(2.85, 7.15)	4.8(2.2, 6.85)	0.3028
GH (cm)	5.15(2.2, 7)	5(3.2, 6.9)	0.6504
CRP (mg/dl)	0.41(0.06, 1.80)	0.54(0.105, 1.84)	0.8294
DAPSA	22.3(14.8, 35.4)	19.7(12.7, 32.6)	0.4010
PASI	4.1(0.925, 10.35)	3.3(0.65, 6.8)	0.4320

Data are shown as mean+/-SD, median (IQR), or n (%), *p <0.05, by Mann-Whitney U test, or Fisher's exact test. NA, not applicable; BMI, body mass index; MTX, methotrexate; PSL, prednisolone; CsA, cyclosporine; SASP, salazosulfapyridine; TJC, tender joint counts; SJC, swollen joints count; UST, ustekinumab; IL-17-i, IL-17 inhibitors; TNF-i, TNF inhibitors; DAPSA, disease activity in PsA; DAPSA-remission(REM) ≦ 4, DAPSA-LDA ≦ 14, PASI, psoriasis area and severity index. Bold values indicated significant differences.

**Table 2:** Comparison of treatment response between 2 groups.

**Variables**	**Standard treatment group (*n =* 56)**	**Strategic treatment group (*n =* 41)**	***p*** **-value**
**Month 6**			
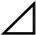 TJC(M6-BL)	−7.75(-3, 0)	−5(-9,−2)	0.0909
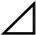 SJC(M6-BL)	−2(-6, 0)	−2(-6,−0.5)	0.3385
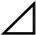 CRP(mg/dl)(M6-BL)	−0.035(-0.9529, 0)	−0.18(-1.675,−0.0015)	0.2037
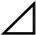 Pain VAS(cm)(M6-BL)	−1.85(-4.95, 0)	−3(-4.15,−0.95)	0.3955
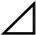 GH(cm)(M6-BL)	−1.45(-3.95, 0)	−2.5(-4.55,−1)	0.1599
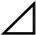 DAPSA(M6-BL)	−9.83(-23.04,−1.65)	−15.7(-24.52,−8.575)	0.1608
Proportion of REM, *n* (%)	25(44.6)	17(41.5)	0.8368
Proportion of REM/LDA, *n* (%)	38(67.8)	37(90.2)	*0.0132
%improvement-PASI	72(0, 100)	100(72.7, 100)	*0.0401
PASI90, *n* (%)	22/47(46.8)	20/36(55.6)	0.5086
Minimal Disease Activity	34(60.7)	33(80.5)	*0.0464
TJC≦1	41(73.2)	31(75.6)	0.8189
SJC≦1	45(80.4)	33(80.5)	1.0000
PASI≦1	42(75.0)	40(97.6)	*0.0032
Patient pain VAS≦15mm	32(57.1)	24(58.5)	1.0000
Patient global disease activity VAS≦20mm	34(60.7)	29(70.7)	0.3901
HAQ score≦0.5	42(75.0)	25(61.0)	0.1829
Tender entheseal points≦1	44(78.5)	31 (75.6)	0.8081
**Month 12**			
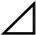 TJC(M12-BL)	−3.5(-6.5, 0)	−4(-9,−2)	0.1632
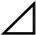 SJC(M12-BL)	−1(-5.75, 0)	−3(-5.5,−0.5)	0.2229
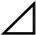 CRP(mg/dl)(M6-BL)	−0.13(-1.32,−0.01)	−0.03(-1.0275, 0)	0.4449
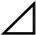 Pain VAS(cm)(M6-BL)	−1(-4.65, 0.075)	−3.2(-4.65,−0.4)	0.1667
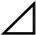 GH(cm)(M12-BL)	−1.685(-4.2, 0)	−2.9(-5.05,−0.65)	0.1783
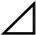 DAPSA	−9.475(-20.56, 0)	−13.94(-26.52,−7.47)	0.1018
Proportion of REM, *n* (%)	23(42.6)	22(53.7)	0.3069
Proportion of REM/LDA, *n* (%)	35(64.8)	35(85.4)	*0.0338
%improvement-PASI	91.5(0, 100)	100(87.5, 100)	*0.0346
PASI90, *n* (%)	23/46(50.0)	26/35(74.2)	*0.0387
Minimal disease activity	30(55.6)	29(70.7)	0.1423
TJC ≦ 1	38(67.9)	29(70.7)	0.8262
SJC ≦1	43(76.8)	34(82.9)	0.6125
PASI≦ 1	40(71.4)	36(87.8)	0.0791
Patient pain VAS ≦ 15 mm	30(53.6)	25(61.0)	0.5364
Patient global disease activity VAS ≦ 20 mm	35(62.5)	24(58.5)	0.8335
HAQ score ≦ 0.5	42(75.0)	23(63.4)	0.2642
Tender entheseal points≦1	44(78.6)	32(78.1)	1.0000

Data are shown as mean+/–SD, median (IQR), or n (%), *p <0.05, by Mann-Whitney U test, or Fisher's exact test. NA, not applicable; MTX, methotrexate; TJC, tender joint counts; SJC, swollen joints count; UST, ustekinumab; IL-17-i, IL-17 inhibitors; TNF-i, TNF inhibitors; DAPSA, disease activity in PsA; DAPSA-remission(REM) ≦4; DAPSA-LDA≦14; PASI, psoriasis area and severity index. Bold values indicated significant differences.

### Retention rate and safety profile

The retention rates and safety profiles are shown in Table 3. In the standard bDMARDs treatment group, the retention rates were 87.5% (49/56 cases) and 82.1% (46/49 cases) at months 6 and 12, respectively. In the strategic bDMARDs treatment group, the retention rates were 100% (41/41 cases) and 95.1% (39/41 cases) at months 6 and 12, respectively. The retention rate at month 6 was significantly higher in the strategic bDMARDs treatment group. The retention rate at month 12 did not differ between the two groups. With respect to the adverse events classified according to the CTCAE, no differences were observed in the total number of events, the number of Grade 3 or higher events, or the number of Grade 2 or higher events.

**Table 3:** Comparison of safety profile.

**Variables**	**Standard treatment group (*n =* 56)**	**Strategic treatment group (*n =* 41)**	***p*** **-value**
**Retention rate**			
*n* (%) (≦6M)	49(87.5)	41(100)	*0.0198
*n* (%) (≦12M)	46(82.1)	39(95.1)	0.0666
**Adverse events**			
Total, *n* (%)	14(66.7)	7(17.7)	0.4561
CTCAE grade 3	herpes-zoster (*n =* 1) pneumocystis pneumonia (*n =* 1),	none	0.5069
CTCAE grade 2	sinusitis (*n =* 1) leg swelling (*n =* 1), upper respiratory infection (*n =* 1) phlegmon (*n =* 1),	Herpers-Zoster (*N =* 1) upper respiratory infection (*n =* 1)	0.4609
CTCAE grade 1	Thrombopenia (*n =* 1), Liver dysfunction (*n =* 3), injection site reaction (*n =* 2), COVID-19(*n =* 1) common cold (*n =* 1)	Thrombocytopenia (*n =* 2), liver dysfunction (*n =* 2), common cold (*n =* 1)	0.4561

Data are shown as n (%), *p < 0.05, by Fisher's exact test. CTCAE, National Cancer Institute Common Terminology Criteria for Adverse Events (CTCAE) version 5.0. Bold values indicated significant differences.

### Impact of BDMARDs treatment on T helper lymphocytic phenotypes

We present in Table 4 the differences in baseline characteristics among the four types (activated Th1-dominant type, activated Th17-dominant type, activated Th1/Th17-hybrid type, and activated Th1/Th17-comparable type in the strategic bDMARDs treatment group at baseline. We differentially and strategically selected the bDMARDs for each group. Accordingly, there was a significant difference in bDMARDS initiation (UST, TNF-i, IL-17-i) and the proportion of aTh1/CD4 (%) and aTh17/CD4 (%). However, there were no differences in the clinical characteristics among the four types.

**Table 4:** Comparison of baseline characteristics among 4 groups.

**Variables**	**Activated Th1-dominant** **(*n =* 5)**	**Activated Th17-dominant** **(*n =* 10)**	**Activated Th1/17–hybrid** **(*n =* 16)**	**Activated Th1/17-comparable** **(*n =* 10)**	***p*-value**
Age (years)	54.8 ± 18.7	52.4 ± 17.2	56.5 ± 14.0	58.6 ± 14.4	0.8380
Male, *n* (%)	3(60.0)	7(70.0)	12(75.0)	3(30.0)	0.1272
**Disease Duration (months)**					
PSO(M)	122(72.5, 318)	72(31, 132)	246(78, 420)	93.5(5.25, 256.3)	0.0914
PsA(M)	7(4, 97.5)	28(2.5, 49.3)	48(12.8, 151.5)	120(6.75, 182.5)	0.1754
Peripheral arthritis, *n* (%)	5(100)	10(100)	16(100)	10(100)	1.0000
Spinal involvement, *n* (%)	0(0)	2(20.0)	5(31.3)	3(30.0)	0.5132
Bio naïve	5(100)	8(80.0)	10(62.5)	8(80.0)	0.3458
Concomitant MTX use	2(40.0)	8(80.0)	6(37.5)	6(60.0)	0.1713
**bDMARDS initiated**					
IL-17-i	0(0)	10(100)	7(43.8)	0(0)	* <0.0001
TNF-i	0(0)	0(0)	9(56.3)	10(100)	* <0.0001
UST	5(100)	0(0)	0(0)	0(0)	* <0.0001
**Disease activity**					
TJC (68)	11(2, 16.5)	6(3.75, 17.3)	5.5(2, 9)	4.5(2, 7.25)	0.5210
SJC (66)	6(1.5, 9)	3(1.5, 6.75)	3(0.25, 5.75)	3.5(1.5, 7.5)	0.7720
Pain VAS (cm)	6.7(1.6, 7.4)	4.35(2.2, 5.33)	5.6(3.13, 7.8)	4.9(0.65, 6.18)	0.7016
GH (cm)	5(3.2, 7.4)	4.05(2.15, 5.3)	6.35(3.5, 7)	4.85(0.78, 6.78)	0.3384
CRP (mg/dl)	0.64(0.11, 1.94)	0.41(0.09, 1.58)	1.02(0.17, 2.20)	0.14(0.04, 2.38)	0.4902
DAPSA	32.8(10.2, 38.7)	21.5(12.9, 35.6)	18.5(14.7, 30.9)	20.4(4.34, 32.9)	0.8108
PASI	6.6(1.75, 11.9)	1(0.3, 6.23)	5.65(1.98, 13.1)	2(0.15, 3.75)	0.0546
aTh1/CD4(%)	2.12(1.70, 2.75)	0.95(0.62, 1.25)	2.11(1.71, 2.23)	0.79(0.43, 1.10)	* <0.0001
aTh17/CD4(%)	1.00(0.76, 1.09)	1.72(1.52, 2.78)	1.76(1.59, 2.17)	0.50(0.43, 0.93)	* <0.0001

Data are shown as mean+/-SD, median (IQR), or n (%), *p <0.05, by Kruskal-Walis test, orchi-square test. NA, not applicable; MTX, methotrexate; TJC, tender joint counts; SJC, swollen joints count; UST, ustekinumab; IL-17-i, IL-17 inhibitors; TNF-i, TNF inhibitors; DAPSA, disease activity in PsA; DAPSA-remission(REM) ≦4, DAPSA-LDA≦14, PASI, psoriasis area and severity index, aTh1, activated Th1 cells, aTh17, activated Th17 cells. Bold values indicated significant differences.

**Figure 2:**
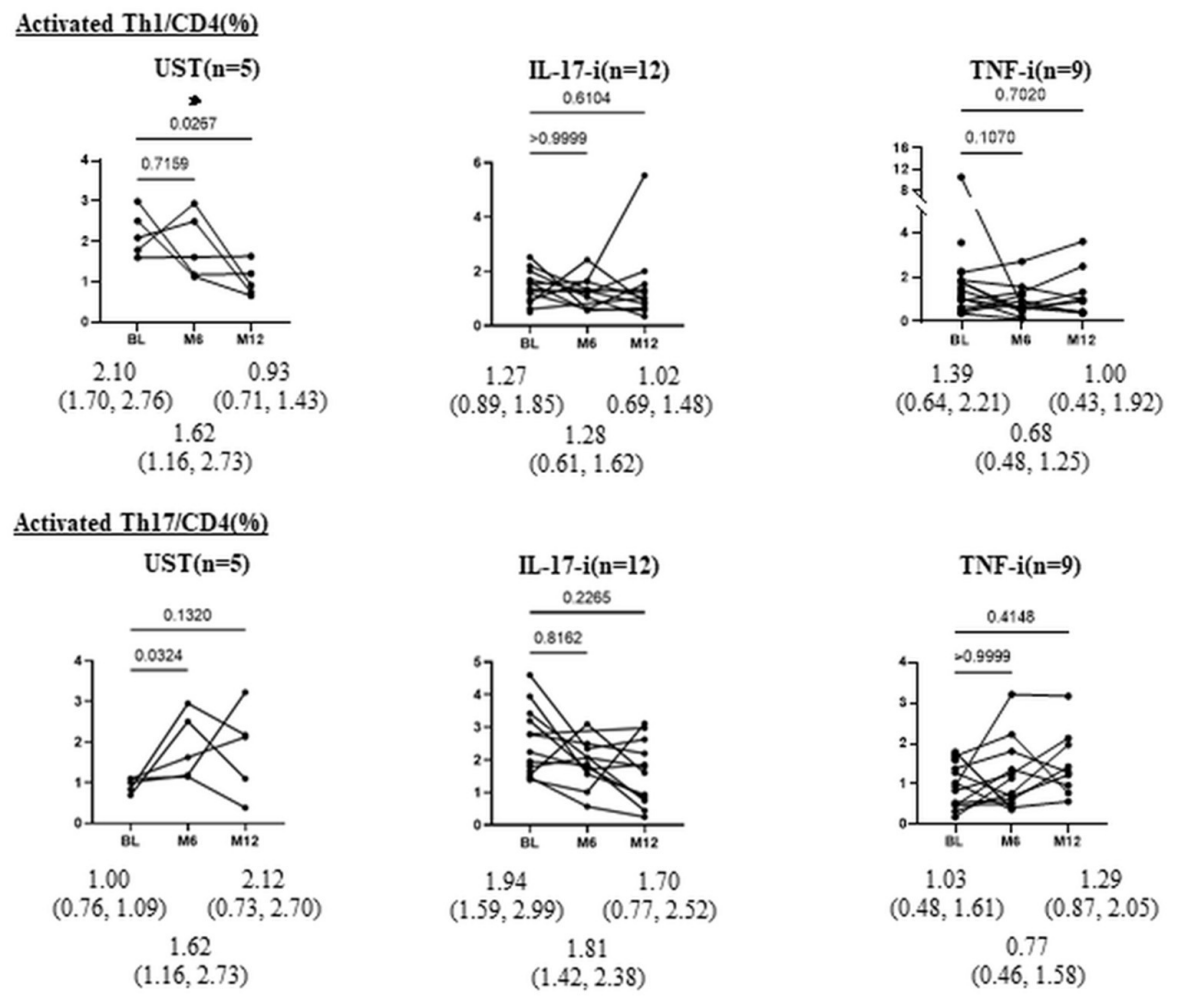
Changes in proportion of aTh1/Th17 cells (%) after bDMARDs treatment. Data are presented as the median (interquartile range [IQR]). *P < 0.05, Wilcoxon signed-rank test. UST, ustekinumab; IL-17-i, IL-17 inhibitors; TNF-i, TNF inhibitors, index, aTh1: activated Th1 cells; aTh17, activated Th17 cells.

**Figure 3:**
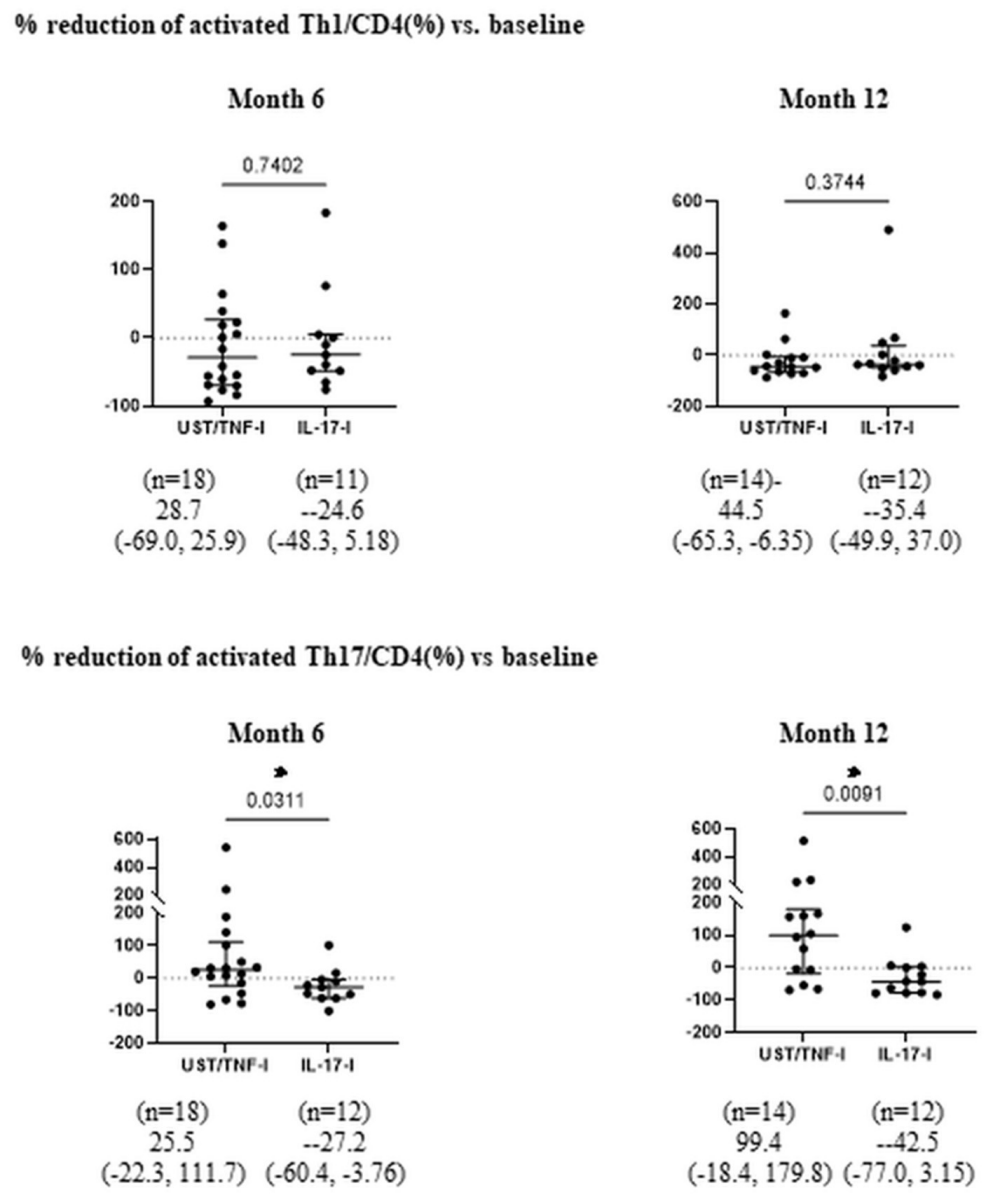
Comparison of percent reduction of aTh1/Th17 cells after bDMARDs treatment. Data are presented as the median (interquartile range [IQR]). *P < 0.05, Mann-Whitney U test. UST, ustekinumab; IL-17-i, IL-17 inhibitors; TNF-i, TNF inhibitors, index, aTh1: activated Th1 cells; aTh17, activated Th17 cells.

Moreover, after treatment with UST, the proportion of aTh1/CD4 (%) significantly decreased at month 12 (Figure 2). There was no significant difference in the proportion of aTh1/CD4 (%) after 1 year of treatment with IL-17-i and TNF-i. In the comparison of the percent reduction of activated Th1/CD4 and activated Th17/CD4, the percent reduction of activated Th17/CD4 was significantly higher in the IL-17-i group at months 6 and 12 (Figure 3). There was no difference in the percent reduction of activated Th1/CD4. We analyzed the correlation between the percent reduction in activated Th1/Th17 and percent improvement in disease activity (DAPSA and PASI) (Supplementary Figure 1). However, we found no correlation between changes in lymphocytic phenotype and disease activity.

## Discussion

This retrospective study validated the effectiveness of precision medicine based on peripheral helper T cell phenotyping during an extended observation period, with DAPSA and MDA selected as treatment targets. In addition, this study demonstrated the impact of bDMARDs treatment on peripheral helper T cell phenotypes in patients with PsA, a highly heterogeneous disease with varying clinical symptoms. We had previously reported the possibility of precision medicine based on phenotyping of peripheral blood (4). However, in that study, we had used SDAI to evaluate the disease activity; moreover, the observation period was as short as 6 months.

In the present study, we extended the observation period to 1 year, selected DAPSA as an indicator of disease activity, and evaluated the rate of achieving MDA at 1 year. Despite the unfavorable conditions in the strategic bDMARDs treatment group (namely, the significantly higher age, smoking history, and the lower rate of concomitant MTX use), the rate of achieving DAPSA-LDA at months 6 and 12 were significantly higher in the strategic bDMARDs treatment group. The rate of achieving MDA at month 6 and PASI90 at month 12 was also significantly higher in the strategic bDMARDs treatment group. We considered that a higher achievement rate of PASI ≦1 contributed to the significantly higher MDA achievement rate at month 6 in the strategic treatment group (Table 2).

Because bDMARDs were strategically selected in the treatment group, the baseline characteristics differed, as shown in Table 1. The proportion of patients treated with IL-17-i was significantly higher in the strategic bDMARDs treatment group, whereas the proportion of patients treated with TNF-i was significantly higher in the standard bDMARDs treatment group. No differences were observed in the proportion of patients treated with UST. Although head-to-head studies comparing TNF-i and IL-17-i have reported comparable efficacy of these inhibitors, at least for joint symptoms (7, 8), we compared the effectiveness of TNF-i and IL-17-i in this cohort in consideration of the possibility that the differences in their effectiveness might have affected the results. As shown in Supplementary Tables 1, 2, neither the strategic nor standard bDMARDs treatment group showed any differences in either clinical characteristics or effectiveness up to month 12 between patients treated with TNF-i and those treated with IL-17-i. TNF-i and IL-17-i were more effective in the strategic bDMARDs treatment group, according to the numerical values. Taken together, the results seem to suggest that the difference in treatment responses between the two groups was not attributable to biased selection toward more effective or less effective drugs. Instead, it is likely that the difference was attributable to the strategies that we established. Although no differences were observed in the rates of achieving either DAPSA-REM or MDA at month 12, these rates at month 6 were significantly higher in the strategic bDMARDs treatment group.

Regarding the retention rate, despite the lack of significant difference at month 12, the retention rate at month 6 was significantly higher in the strategic bDMARDs treatment group (Table 3), which neither include non-responders nor patients who discontinued treatment because of adverse events (Figure 1). This suggests that our strategies might contribute to favorable treatment responses and treatment retention, especially because of the safety profile immediately after the initiation of bDMARDs treatment and the preventive effect on transient ineffectiveness. Given that the biased selection of drugs might also have contributed to the difference in the retention rates between the two groups, we compared the retention rates between patients treated with TNF-i and those treated with IL-17-i in the standard bDMARDs treatment group, but no difference was observed between the retention rates of the two groups treated with different drugs (Supplementary Table 3). Further study with a longer observation period is essential to determine the long-term treatment efficacy and safety.

In patients who underwent peripheral helper T-lymphocyte phenotyping at months 6 and 12, we also evaluated changes in phenotypes after treatment (Figure 2). There were no statistically significant differences in the proportion of activated Th1 or Th17 cells (%) at baseline between patients with or without spinal involvement (Supplementary Table 4). The results revealed a significant decrease in the proportion of activated Th1 cells in patients treated with UST.

We hypothesized that the IL-12/23 (p40) inhibitor UST predominantly acts on Th1 in the dendritic cell-Th1 or Th17 pathway and, thus, strategically selected UST for the activated Th1 cell-dominant type. This decrease supports the validity of our hypotheses. In patients treated with IL-17-i or TNF-i, the proportion of activated Th1/Th17 cells did not change. Given that IL-17-i is a drug that inhibits IL-17, which is an effector cytokine for Th17, but does not directly inhibit Th17 differentiation, the effect of the inhibitor on activated Th17 seems to have been unremarkable. When the rate of decrease in activated Th17 cells was compared between patients treated with IL-17-i and those treated with UST or TNF-i, the rate was found to be significantly higher in patients treated with IL-17-i. IL-17 inhibits Th1 cell differentiation by inhibiting the expression of IL-12 receptors on naïve T-cells (9). Thus, IL-17-i reversed the inhibition of IL-12 receptor expression (enhanced expression) and induced Th1 cell differentiation. Consequently, the rate of decrease in activated Th17 cells was higher for IL-17-i than for UST/TNF-i cells. However, the number of patients, particularly in the UST treated group, is quite small, so it is essential to conduct further studies with larger sample sizes.

Although the effects on helper T cell phenotypes may vary among drugs, as described above, our results indicate that TNF-i and IL-17-i were comparable in effectiveness, as shown in Supplementary Tables 1, 2. Moreover, treatment responses were not significantly correlated with changes in lymphocytic phenotypes (Supplementary Figure 1). It is likely that lymphocytic phenotypes do not simply reflect disease activity but also the treatment targets for individual patients. An investigation of the changes in lymphocytic phenotypes and the impact on other immune cells in patients receiving treatments that differ from the strategy we report here might contribute to a more detailed picture of the pathology of PsA and, consequently, the establishment of more efficient strategies.

Recent reports have also shown the effectiveness of IL-23(p19)-i and JAK-i in PsA (10, 11). In the present study, we excluded IL-23(p19)-i and JAK-i because these drugs were recently launched in our country, and we have not collected sufficient clinical data that can be analyzed. Considering its mode of action, Il-23(p19)-i is assumed to be more effective in the activated Th17 cell-dominant subtype. Efficacy of JAK-i could differ depend on its selectivity. It is necessary to conduct studies on the effectiveness of these new drugs and their impact on the lymphocytic phenotypes.

## Conclusions

With this retrospective study, we have demonstrated the 1-year effectiveness of precision medicine based on peripheral T-lymphocytic phenotyping in terms of DAPSA and MDA. Data from real-world clinical practice reveal that the impact on the immune system varied among bDMARDs. However, there are possibilities that landscapes of the treat to target, and/or biases affected the outcomes. In addition, because PsA is a disease with very high heterogeneity, it is necessary to conduct studies with larger sample sizes drawing from multiple institutions.

## Data availability statement

The original contributions presented in the study are included in the article/Supplementary material, further inquiries can be directed to the corresponding author/s.

## Ethics statement

The studies involving human participants were reviewed and approved by University of Occupational and Environmental Health, Japan Ethics Committee (Approval Number #04-23). The patients/participants provided their written informed consent to participate in this study.

## Author contributions

IM contributed to the study design, overall review, and writing of the manuscript, while the other authors were involved in the performance of the study and review of the manuscript. MU, YM, NO, and YI contributed to the flow cytometry analysis. YT, SN, SK, and YM participated in study design and coordination. All the authors have read and approved the final version of the manuscript.

## Funding

This work was supported in part by Research on rare and intractable diseases and Research Grant-In-Aid for Scientific Research by the Ministry of Health, Labor and Welfare of Japan, the Ministry of Education, Culture, Sports, Science and Technology of Japan; the Japan Agency for Medical Research and Development; International Research Fund for Subsidy of Kyushu University School of Medicine Alumni, and the University of Occupational and Environmental Health, Japan; and UOEH Grant for Advanced Research (#19K17919).

## Conflict of interest

YT has received speaking fees and/or honoraria from Daiichi-Sankyo, Eli Lilly, Novartis, YL Biologics, Bristol-Myers, Eisai, Chugai, Abbvie, Astellas, Pfizer, Sanofi, Asahi-kasei, GSK, Mitsubishi-Tanabe, Gilead, and Janssen; research grants from Abbvie, Mitsubishi-Tanabe, Chugai, Asahi-Kasei, Eisai, Takeda, Daiichi-Sankyo; and consultant fees from Eli Lilly, Daiichi-Sankyo, Taisho, Ayumi, Sanofi, GSK, and Abbvie. SN has received consulting fees, speaking fees, and/or honoraria from Bristol-Myers, AstraZeneca, Pfizer, GlaxoSmithKline, Astellas, Asahi-kasei, Sanofi, Abbvie, Eisai, Chugai, Gilead, and Boehringer Ingelheim, and has received research grants from Mitsubishi-Tanabe. YM has received consulting fees, speaking fees, and/or honoraria from Eli Lilly, and has received research grants from GlaxoSmithKline. The remaining authors declare that the research was conducted in the absence of any commercial or financial relationships that could be construed as a potential conflict of interest.

## Publisher's note

All claims expressed in this article are solely those of the authors and do not necessarily represent those of their affiliated organizations, or those of the publisher, the editors and the reviewers. Any product that may be evaluated in this article, or claim that may be made by its manufacturer, is not guaranteed or endorsed by the publisher.

## Supplementary material

Supplementary Table 1 Comparison of baseline characteristics and treatment response between TNF-i and IL-17-i in strategic treatment group. Data are shown as mean +/−SD, median (IQR), or n (%), ^*^p < 0.05, by Mann-Whitney U test, or Fisher's exact test. NA, not applicable; MTX, methotrexate; TJC, tender joint counts; SJC, swollen joints count; IL-17-i, IL-17 inhibitors; TNF-i, TNF inhibitors; DAPSA, disease activity in PsA; DAPSA-remission(REM) ≦4; DAPSA-LDA≦14; PASI, psoriasis area and severity index.

Supplementary Table 2 Comparison of baseline characteristics and treatment response between TNF-i and IL-17-i in standard treatment group. Data are shown as mean+/−SD, median (IQR), or n (%), ^*^p < 0.05, by Mann-Whitney U test, or Fisher's exact test. NA, not applicable. MTX, methotrexate; TJC, tender joint counts; SJC, swollen joints count; IL-17-i, IL-17 inhibitors; TNF-i, TNF inhibitors; DAPSA, disease activity in PsA; DAPSA-remission(REM) ≦4; DAPSA-LDA≦14; PASI, psoriasis area and severity index.

Supplementary Table 3 Comparison of retention rate between TNF-i and IL-17-i in standard treatment group. Data are shown as n (%), ^*^p < 0.05, by Fisher's exact test. CTCAE, National Cancer Institute Common Terminology Criteria for Adverse Events (CATAE) version 5.0.

Supplementary Table 4 The proportion of activated Th1 or Th17 cells (%) in patients with and without spinal involvement. Median (IQR), by Mann-Whitney U test. Median (IQR), by Mann-Whitney U test.
