# Supplementary material for: Precision medicine based on the phenotypic differences in peripheral T helper cells in patients with psoriatic arthritis: One year follow-up outcomes
Source: Front Med (Lausanne). 2022 Jul 27;9:934937. doi: 10.3389/fmed.2022.934937 (PMC9363692; doi:10.3389/fmed.2022.934937)
Supplement: Supplementary Table 1 — Comparison of baseline characteristics and treatment response between TNF-i and IL-17-i in strategic treatment group. Data are shown as mean +/−SD, median (IQR), or n (%), *p < 0.05, by Mann-Whitney U test, or Fisher's exact test. NA, not applicable; MTX, methotrexate; TJC, tender joint counts; SJC, swollen joints count; IL-17-i, IL-17 inhibitors; TNF-i, TNF inhibitors; DAPSA, disease activity in PsA; DAPSA-remission(REM) ≦4; DAPSA-LDA≦14; PASI, psoriasis area and severity index. [file Table_1.DOCX]

| variables | **TNF-i (n=19)** | **IL-17-i (n=17)** | **p-value** |
| --- | --- | --- | --- |
| ***Baseline characteristics*** |  |  |  |
| **Age (years)** | 60.0±12.5 | 51.5±14.6 | 0.0614 |
| **Male, n (%)** | 9(47.4) | 13(76.5) | 0.0967 |
| **Disease Duration (months)** |  |  |  |
| **PSO(M)** | 168(10, 305) | 108(48, 300) | 0.9116 |
| **PsA(M)** | 48(12, 168) | 32(5, 71.5) | 0.1776 |
| **Peripheral arthritis, n (%)** | 19(100) | 17(100) | 1.0000 |
| **Spinal involvement, n (%)** | 5(26.3) | 5(29.4) | 1.0000 |
| **Bio naïve** | 16(82.4) | 10(58.9) | 0.1394 |
| **Concomitant MTX use** | 9(47.4) | 7(41.2) | 0.7486 |
| **Disease activity** |  |  |  |
| **TJC (68)** | 5(2, 7) | 6(2.5, 10) | 0.6673 |
| **SJC (66)** | 3(1, 6) | 3(0.5, 7) | 0.9618 |
| **Pain VAS (cm)** | 4.8(2, 6.7) | 4.7(2.2, 6.75) | 0.9747 |
| **GH (cm)** | 4.8(3.5, 6.8) | 5(2.2 ,6.85) | 0.7628 |
| **CRP (mg/dl)** | 0.54(0.06, 2.03) | 0.53(0.12, 1.70) | 0.8991 |
| **DAPSA** | 18.7(10.4, 26.7) | 21.1(12.8, 33.0) | 0.5577 |
| **PASI** | 3.3(0.6, 5.7) | 1.6(0.6, 10.8) | 0.9241 |
| ***Treatment response*** |  |  |  |
| **(Month 6)** |  |  |  |
| **⊿TJC(M6-BL)** | -4(-6, -2) | -6(-9, -2.5) | 0.4724 |
| **⊿SJC(M6-BL)** | -3(-5, -1) | -2(-5.5, -0.5) | 0.7486 |
| **⊿CRP(mg/dl)(M6-BL)** | -0.43(-2.01, -0.02) | -0.07(-1.40, 0.03) | 0.2605 |
| **⊿Pain VAS(cm)(M6-BL)** | -3(-4, -0.1) | -3.1(-4.55, -1.4) | 0.6342 |
| **⊿GH(cm)(M6-BL)** | -2.6(-5.2, -0.1) | -3(-4.8, -1.2) | 0.4563 |
| **⊿DAPSA(M6-BL)** | -10.4(-24.6, -8.51) | -15.7(-24.3, -9.97) | 0.4957 |
| **Proportion of REM, n(%)** | 9(47.4) | 6(35.3) | 0.5160 |
| **Proportion of REM/LDA, n(%)** | 18(94.8) | 16(94.1) | 1.0000 |
| **%improvement-PASI** | 86.8(54.1, 100) | 100(43.1, 100) | 0.6628 |
| **PASI90, n(%)** | 10/16(62.5) | 7/15(47.7) | 0.4795 |
| **Minimal Disease Activity** | 16(84.2) | 14(82.4) | 1.0000 |
|  |  |  |  |
| **(Month 12)** |  |  |  |
| **⊿TJC(M12-BL)** | -3(-7, -2) | -6(-9, -1.5) | 0.3723 |
| **⊿SJC(M12-BL)** | -3(-6.5, -0.5) | -2(-5, 0) | 0.6776 |
| **⊿CRP(mg/dl)(M12-BL)** | -0.13(-1.71, -0.03) | -0.04(-1.28, 0.05) | 0.3663 |
| **⊿Pain VAS(cm)(M12-BL)** | -2.4(-4, 0) | -4(-4.7, -1.46) | 0.1717 |
| **⊿GH(cm)(M12-BL)** | -2.5(-5.5, 0) | -3.2(-5.05, -0.75) | 0.4659 |
| **⊿DAPSA** | -12.4(-24.0, -3.62) | -14.9(-27.5, -8.28) | 0.2884 |
| **Proportion of REM, n(%)** | 11(57.9) | 9(52.4) | 1.0000 |
| **Proportion of REM/LDA, n(%)** | 15(78.9) | 16(94.1) | 0.3420 |
| **%improvement-PASI** | 100(68.6, 100) | 100(88.3, 100) | 0.4980 |
| **PASI90, n (%)** | 11/16(68.8) | 10/15(66.7) | 1.0000 |
| **Minimal Disease Activity** | 14(73.7) | 13(76.5) | 1.0000 |

**Supplemental　Table 1. Comparison of baseline characteristics and treatment response between TNF-i and IL-17-i in strategic treatment group**

Data are shown as mean+/-SD, median (IQR), or n (%), * p<0.05, by Mann-Whitney U test, or Fisher’s exact test. NA: not applicable. MTX: methotrexate, TJC: tender joint counts, SJC: swollen joints count, IL-17-i: IL-17 inhibitors, TNF-i: TNF inhibitors, DAPSA: disease activity in PsA, DAPSA-remission(REM) ≦4, DAPSA-LDA≦14, PASI: psoriasis area and severity index
