# Supplementary material for: Precision medicine based on the phenotypic differences in peripheral T helper cells in patients with psoriatic arthritis: One year follow-up outcomes
Source: Front Med (Lausanne). 2022 Jul 27;9:934937. doi: 10.3389/fmed.2022.934937 (PMC9363692; doi:10.3389/fmed.2022.934937)
Supplement: Supplementary Table 2 — Comparison of baseline characteristics and treatment response between TNF-i and IL-17-i in standard treatment group. Data are shown as mean+/−SD, median (IQR), or n (%), *p < 0.05, by Mann-Whitney U test, or Fisher's exact test. NA, not applicable. MTX, methotrexate; TJC, tender joint counts; SJC, swollen joints count; IL-17-i, IL-17 inhibitors; TNF-i, TNF inhibitors; DAPSA, disease activity in PsA; DAPSA-remission(REM) ≦4; DAPSA-LDA≦14; PASI, psoriasis area and severity index. [file Table_2.DOCX]

| **variables** | **TNF-i (n=40)** | **IL-17-i (n=7)** | **p-value** |
| --- | --- | --- | --- |
| ***Baseline characteristics*** |  |  |  |
| **Age (years)** | 60.0±12.5 | 51.5±14.6 | 0.0614 |
| **Male, n (%)** | 9(47.4) | 13(76.5) | 0.0967 |
| **Disease Duration (months)** |  |  |  |
| **PSO(M)** | 168(10, 305) | 108(48, 300) | 0.9116 |
| **PsA(M)** | 48(12, 168) | 32(5, 71.5) | 0.1776 |
| **Peripheral arthritis, n (%)** | 19(100) | 17(100) | 1.0000 |
| **Spinal involvement, n (%)** | 5(26.3) | 5(29.4) | 1.0000 |
| **Bio naïve** | 16(82.4) | 10(58.9) | 0.1394 |
| **Concomitant MTX use** | 9(47.4) | 7(41.2) | 0.7486 |
| **Disease activity** |  |  |  |
| **TJC (68)** | 5(2, 7) | 6(2.5, 10) | 0.6673 |
| **SJC (66)** | 3(1, 6) | 3(0.5, 7) | 0.9618 |
| **Pain VAS (cm)** | 4.8(2, 6.7) | 4.7(2.2, 6.75) | 0.9747 |
| **GH (cm)** | 4.8(3.5, 6.8) | 5(2.2 ,6.85) | 0.7628 |
| **CRP (mg/dl)** | 0.54(0.06, 2.03) | 0.53(0.12, 1.70) | 0.8991 |
| **DAPSA** | 18.7(10.4, 26.7) | 21.1(12.8, 33.0) | 0.5577 |
| **PASI** | 3.3(0.6, 5.7) | 1.6(0.6, 10.8) | 0.9241 |
| ***Treatment response*** |  |  |  |
| **(Month 6)** |  |  |  |
| **⊿TJC(M6-BL)** | -3(-9, 0) | -3(-5, 0) | 0.5962 |
| **⊿SJC(M6-BL)** | -2(-8.75, 0) | -2(-4, 0) | 0.8675 |
| **⊿CRP(mg/dl)(M6-BL)** | -0.09(-1.00, 0) | -0.13(-0.82, 0) | 0.8095 |
| **⊿Pain VAS(cm)(M6-BL)** | -1.8(-4.78, 0) | -2(-7.1, 0) | 0.3603 |
| **⊿GH(cm)(M6-BL)** | -1.5(-4.1, 0) | -1.2(-4.5, 0) | 0.9282 |
| **⊿DAPSA(M6-BL)** | -11.4(-28.5, -1.08) | -5.33(-20.2, -4.13) | 0.9523 |
| **Proportion of REM, n(%)** | 21(52.5) | 2(28.6) | 0.4158 |
| **Proportion of REM/LDA, n(%)** | 28(70) | 6(85.7) | 0.6549 |
| **%improvement-PASI** | 72.0(0, 100) | 100(100, 100) | ***0.0431** |
| **PASI90, n(%)** | 15/34(44.1) | 4/4(100) | 0.1050 |
| **Minimal Disease Activity** | 27(67.5) | 5(57.1) | 0.6758 |
|  |  |  |  |
| **(Month 12)** |  |  |  |
| **⊿TJC(M12-BL)** | -4(-8.75, 0) | -3(-5, 0) | 0.6591 |
| **⊿SJC(M12-BL)** | -1.5(-8.75, 0) | -2(-4, 0) | 0.9157 |
| **⊿CRP(mg/dl)(M12-BL)** | -0.03(-1.03, 0.01) | -0.03(-0.85, 0) | 0.8456 |
| **⊿Pain VAS(cm)(M12-BL)** | -0.45(-4.48, 0.25) | -2(-7.1, 0) | 0.3525 |
| **⊿GH(cm)(M12-BL)** | -1(-4.05, 0) | -1.2(-4.9, 0.4) | 0.9282 |
| **⊿DAPSA** | -11.2(-25.0, 0) | -5.19(-19.5, -2.03) | 0.8342 |
| **Proportion of REM, n(%)** | 19(47.5) | 2(28.6) | 0.4364 |
| **Proportion of REM/LDA, n(%)** | 25(62.5) | 6(85.7) | 0.3956 |
| **%improvement-PASI** | 89.0(0, 100) | 100(100, 100) | 0.0531 |
| **PASI90, n (%)** | 16/34(47.1) | 4/4(100) | 0.1071 |
| **Minimal Disease Activity** | 24(60) | 3(42.9) | 0.4380 |

**Supplemental　Table 2. Comparison of baseline characteristics and treatment response between TNF-i and IL-17-i in standard treatment group**

Data are shown as mean+/-SD, median (IQR), or n (%), * p<0.05, by Mann-Whitney U test, or Fisher’s exact test. NA: not applicable. MTX: methotrexate, TJC: tender joint counts, SJC: swollen joints count, IL-17-i: IL-17 inhibitors, TNF-i: TNF inhibitors, DAPSA: disease activity in PsA, DAPSA-remission(REM) ≦4, DAPSA-LDA≦14, PASI: psoriasis area and severity index
