# Supplementary material for: Precision medicine based on the phenotypic differences in peripheral T helper cells in patients with psoriatic arthritis: One year follow-up outcomes
Source: Front Med (Lausanne). 2022 Jul 27;9:934937. doi: 10.3389/fmed.2022.934937 (PMC9363692; doi:10.3389/fmed.2022.934937)
Supplement: Supplementary Table 3 — Comparison of retention rate between TNF-i and IL-17-i in standard treatment group. Data are shown as n (%), *p < 0.05, by Fisher's exact test. CTCAE, National Cancer Institute Common Terminology Criteria for Adverse Events (CATAE) version 5.0. [file Table_3.DOCX]

| **variables** | **TNF-i (n=40)** | **IL-17-i (n=7)** | **p-value** |
| --- | --- | --- | --- |
| **Retention rate** |  |  |  |
| **n (%) (≦6M)** | 35(87.5) | 7(100) | 0.5728 |
| **n (%) (≦12M)** | 33(82.5) | 7(100) | 1.0000 |

**Supplemental Table 3. Comparison of retention rate between TNF-i and IL-17-i in standard treatment group**

Data are shown as n (%), * p<0.05, by Fisher’s exact test. CTCAE: National Cancer Institute Common Terminology Criteria for Adverse Events (CATAE) version 5.0.
