# Supplementary material for: Precision medicine based on the phenotypic differences in peripheral T helper cells in patients with psoriatic arthritis: One year follow-up outcomes
Source: Front Med (Lausanne). 2022 Jul 27;9:934937. doi: 10.3389/fmed.2022.934937 (PMC9363692; doi:10.3389/fmed.2022.934937)
Supplement: Supplementary Table 4 — The proportion of activated Th1 or Th17 cells (%) in patients with and without spinal involvement. Median (IQR), by Mann-Whitney U test. Median (IQR), by Mann-Whitney U test. [file Table_4.DOCX]

|  | **Spinal involvement (n=10)** | **Without (n=31)** | **p-value** |
| --- | --- | --- | --- |
| **Activated Th1 (%)** | 1.376(0.635, 1.825) | 1.609(0.965, 2.197) | 0.5437 |
| **Activated Th17 (%)** | 1.636(0.821, 2.948) | 1.441(0.944, 1.796) | 0.4124 |

**Supplementary Table 4. The proportion of activated Th1 or Th17 cells (%) in patients with and without spinal involvement.** Median (IQR), by Mann-Whitney U test.
