# Supplementary figures and images for: Precision medicine based on the phenotypic differences in peripheral T helper cells in patients with psoriatic arthritis: One year follow-up outcomes
Source: Front Med (Lausanne). 2022 Jul 27;9:934937. doi: 10.3389/fmed.2022.934937 (PMC9363692; doi:10.3389/fmed.2022.934937)

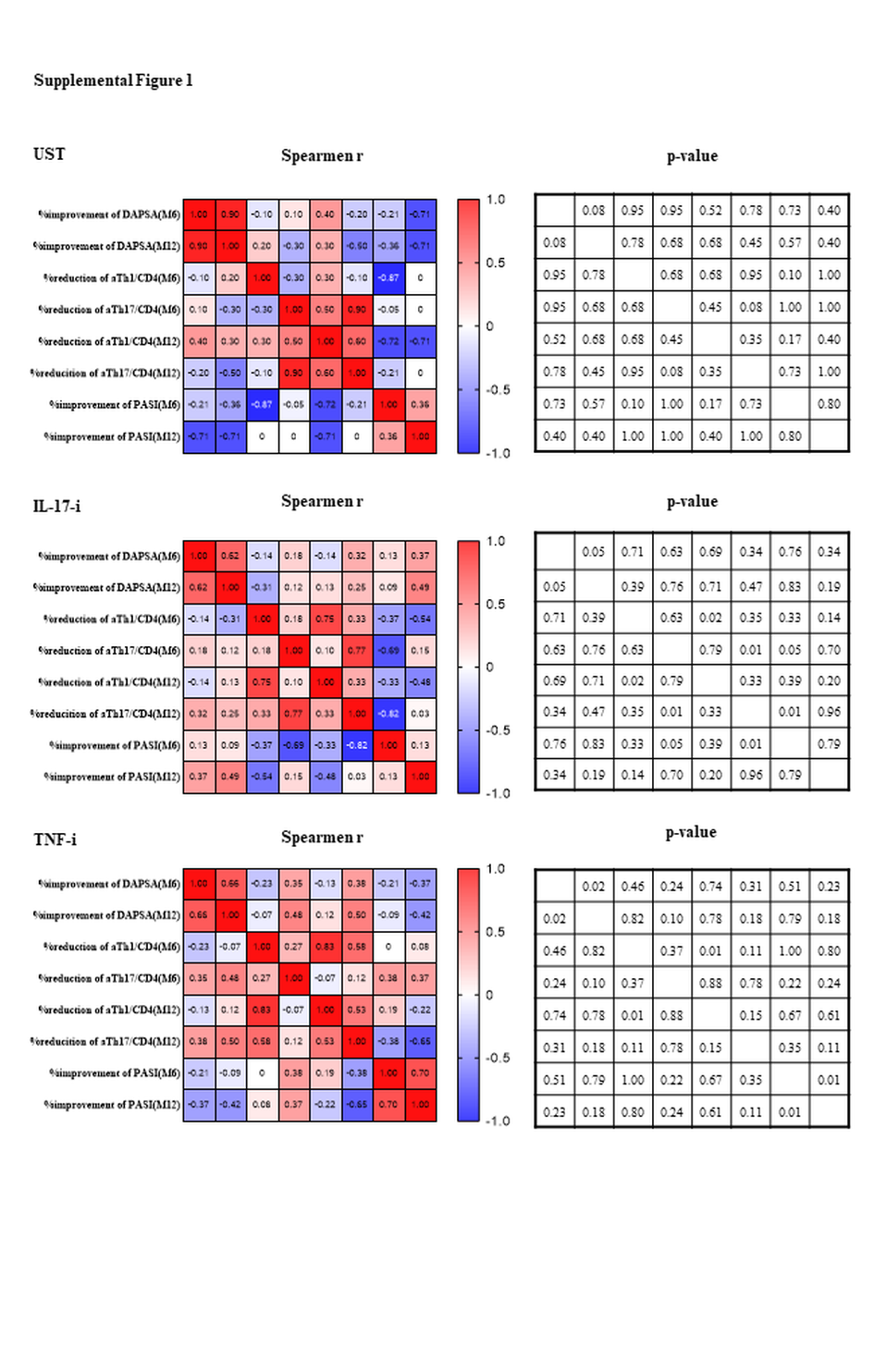

Supplement: Supplementary file 5 [file Image_1.TIF]
